# Expression of Immunotherapy Target PRAME in Cancer Correlates with Histone H3 Acetylation and Is Unrelated to Expression of Methylating (DMNT3A/3B) and Demethylating (TET1) Enzymes

**DOI:** 10.3390/jcm13061554

**Published:** 2024-03-08

**Authors:** Maciej Kaczorowski, Jerzy Lasota, Krzysztof Dudek, Bartosz Małkiewicz, Markku Miettinen, Agnieszka Hałoń

**Affiliations:** 1 Department of Clinical and Experimental Pathology, Wroclaw Medical University, 50-556 Wrocław, Poland; agnieszka.halon@umw.edu.pl; 2 Laboratory of Pathology, National Cancer Institute, Bethesda, MD 20892, USA; jerzy.lasota@nih.gov (J.L.); markku.miettinen@nih.gov (M.M.); 3 Faculty of Mechanical Engineering, Wroclaw University of Science and Technology, 50-370 Wrocław, Poland; krzysztof.dudek@pwr.edu.pl; 4 University Center of Excellence in Urology, Department of Minimally Invasive and Robotic Urology, Wroclaw Medical University, 50-556 Wrocław, Poland; bartosz.malkiewicz@umw.edu.pl

**Keywords:** PRAME, TET1, DNMT3A, DNMT3B, 5hmC, H3ac, seminoma, synovial sarcoma, sinonasal melanoma, methylation, histone acetylation

## Abstract

**Background/Objectives:** Preferentially expressed antigen in melanoma (PRAME), a member of the cancer testis antigen family, is a promising target for cancer immunotherapy. Understanding the epigenetic mechanisms involved in the regulation of PRAME expression might be crucial for optimizing anti-PRAME treatments. **Methods:** Three malignancies of different lineages (sinonasal melanoma, testicular seminoma, and synovial sarcoma), in which immunohistochemical (IHC) reactivity for PRAME is a common yet variable feature, were studied. The expression of PRAME, ten-eleven translocation demethylase 1 (TET1), and DNA methyltransferase (DNMT) 3A and 3B were evaluated using immunohistochemistry. Moreover, the expression of two epigenetic marks, 5-hydroxymethylcytosine (5hmC) and histone 3 acetylation (H3ac), was tested. **Results:** All PRAME-positive tumors expressed medium-to-high levels of H3ac but differed considerably with respect to other markers. In seminomas, PRAME expression correlated with TET1, but in melanomas and synovial sarcomas, it correlated with both DNMTs and DNMT3A, respectively. **Conclusions:** PRAME expression was not determined by a balance between the global expression of DNA methylating/demethylating enzymes. However, histone acetylation may be one of the epigenetic mechanisms involved in PRAME regulation. Thus, the therapeutic combination of histone deacetylase inhibitors and PRAME immunotherapy merits further investigation.

## 1. Introduction

Preferentially expressed antigen in melanoma (PRAME) belongs to the family of cancer testis antigens. In normal tissues, PRAME expression is essentially restricted to the testis and the endometrium; however, it has been detected in a broad spectrum of malignancies [1]. PRAME is a transcriptional repressor of retinoic acid signaling and thereby regulates cellular growth, differentiation, and apoptosis [2]. Moreover, it has been implicated in the promotion of invasion, metastasis, epithelial-to-mesenchymal transition, and genomic instability in a number of cancers [3,4,5,6,7]. Notably, cytotoxic T lymphocytes have been shown to effectively target PRAME-expressing cancer cells [8,9]. Several clinical trials are underway to evaluate the efficacy of PRAME-directed therapies (ClinicalTrials.gov). Understanding the epigenetic mechanisms, such as DNA methylation, that regulate PRAME expression is essential for the optimization of such therapies [10,11].

Methylated DNA forms a dynamic landscape modified by DNA methyltransferases (DNMTs) and ten-eleven translocation (TET) demethylases [12]. DNMT3A and DNMT3B catalyze the de novo methylation of previously unmethylated CpG sites. Conversely, TET enzymes oxidize 5-methylcytosine to 5-hydroxymethylcytosine (5hmC), which can ultimately result in the restoration of unmethylated cytosine [12]. TET1 is instrumental in the demethylation of gene promoters and transcription start sites [13,14]. Dysregulation of DNMTs and TETs has been reported in cancer [12].

Apart from DNA methylation, in vitro experiments suggested a functional association between PRAME expression and histone acetylation [15,16]. This epigenetic mechanism, regulated by the interplay of histone acetyltransferases and deacetylases, relaxes chromatin structure and facilitates gene transcription [17]. Modulation of either DNA methylation or histone 3 acetylation (H3ac) might therefore prove useful in augmenting PRAME expression in the context of anti-PRAME immunotherapy. Both epigenetic processes can be targeted by an expanding family of epigenetic drugs, some of which are already in clinical use [17].

This study aimed to investigate the potential relationship between the expression of PRAME and methylation-regulating enzymes. Moreover, we sought to verify whether PRAME levels are associated with histone acetylation. Three malignancies from different lineages (melanoma, seminoma, and synovial sarcoma), characterized by frequent yet variable PRAME expression [1], were studied. We examined whether the variability of PRAME immunoreactivity is attributable to the differential expression of epigenetic modifiers DNMT3A, DNMT3B, and TET1, as well as levels of 5hmC and H3ac.

## 2. Material and Methods

### 2.1. Tissue Samples

This study evaluated 247 formalin-fixed paraffin-embedded (FFPE) malignant tumors, including 79 testicular seminomas (63 pure and 16 mixed with non-seminomatous components), 66 sinonasal melanomas, and 102 synovial sarcomas. Paraffin blocks with mixed germ cell tumors were dissected to obtain fragments containing seminoma tissues. Samples were arranged in multi-tissue blocks as previously described [18]. Blocks with melanoma and synovial sarcoma samples were previously developed [19,20]. Representative images of the studied tumors are shown in Supplemental Figure S1. The study was carried out in accordance with the Declaration of Helsinki and was approved by the Bioethics Committee of Wroclaw Medical University (Approval #: KB165/2023).

### 2.2. Immunohistochemistry and Evaluation of Staining

Immunohistochemistry (IHC) was performed using either the DAKO or Leica (Leica Biosystems, Bannockburn, IL, USA) platform and panel of antibodies to PRAME, TET1, DNMT3A, DNMT3B, 5hmC, and H3ac (pan-acetyl). Detailed information about antibodies and staining protocols are provided in Supplemental Table S1. Immunoreactivity in neoplastic cells was estimated using histochemical scoring (h-score) assessment. A percentage of stained tumor nuclei multiplied by staining intensity score (0: none; 1: weak; 2: medium; 3: strong) produced a final h-score value ranging from 0 to 300. To exclude potential artifacts from non-specific staining, h-scores ≤ 10 were considered negative.

### 2.3. Statistical Analysis

To assess the correlations between IHC expression of PRAME, the methylation regulators TET1 and DNMT3A/B, and the epigenetic marks 5hmC and H3ac, the values of Kendall’s tau correlation coefficient were estimated. The difference in PRAME expression between seminomas and seminoma components of mixed GCTs was verified with the Mann–Whitney U test. Differences in IHC marker expression between tumor types were analyzed using the Kruskal–Wallis test. The Dunn’s test and Holm–Bonferroni correction were used as post hoc tests. Differences were considered significant when *p* < 0.05. The analysis was conducted using Statistica v.13. (TIBCO Sotfware Inc., Palo Alto, CA, USA).

## 3. Results

### 3.1. Expression of PRAME, Methylation Regulators and Epigenetic Marks

Only the staining of neoplastic cells was evaluated for the purpose of immunohistochemistry scoring. However, some non-neoplastic cells also demonstrated the expression of the analyzed markers and, when present, could be used as internal positive controls. All of the studied markers were expressed by the seminiferous epithelium, albeit with different distributions among cells in various stages of spermatogenesis. 5hmC was also expressed by Leydig cells and some lymphoid cells, mostly weakly, as well as some spindled stromal cells. Similar weak staining of some lymphoid cells was observed in the case of DNMT3A. Scattered-to-diffuse expression of both DNMTs, as well as 5hmC, was present in transitional and squamous epithelium within specimens of mucosal melanomas.

**Figure 1:**
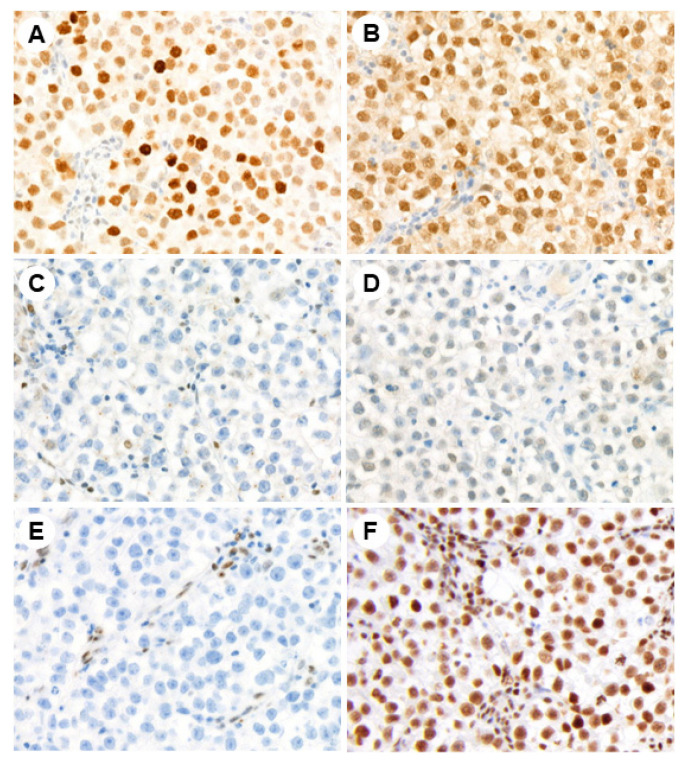
Immunohistochemical expression of PRAME (**A**), TET1 (**B**), DNMT3A (**C**), DNMT3B (**D**), 5hmC (**E**), and H3ac (**F**) in testicular seminoma (magnification ×200).

**Figure 2:**
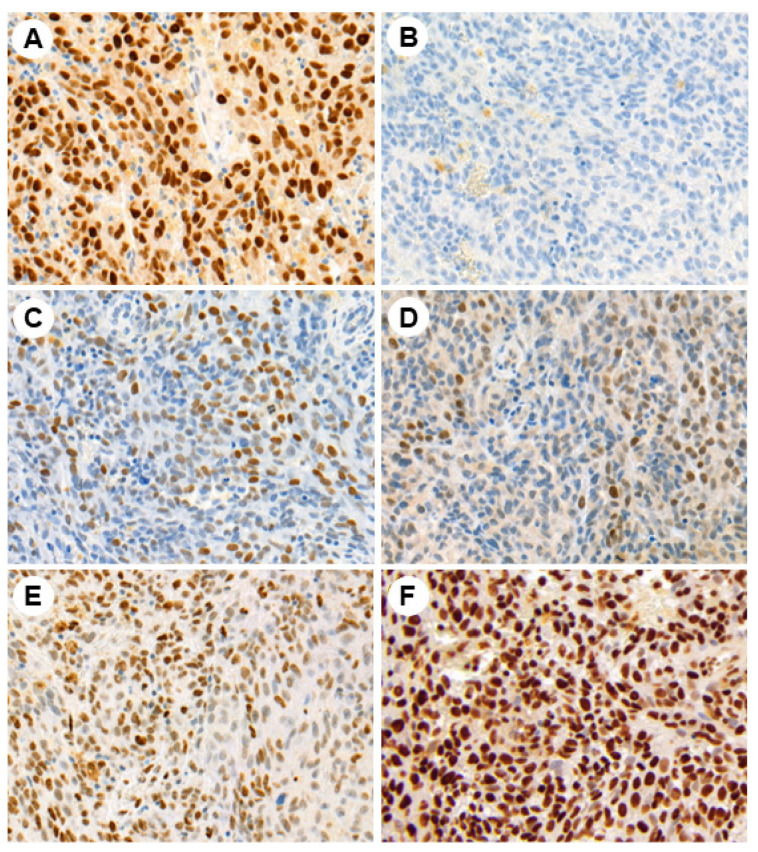
Immunohistochemical expression of PRAME (**A**), TET1 (**B**), DNMT3A (**C**), DNMT3B (**D**), 5hmC (**E**), and H3ac (**F**) in mucosal melanoma (magnification ×200).

**Figure 3:**
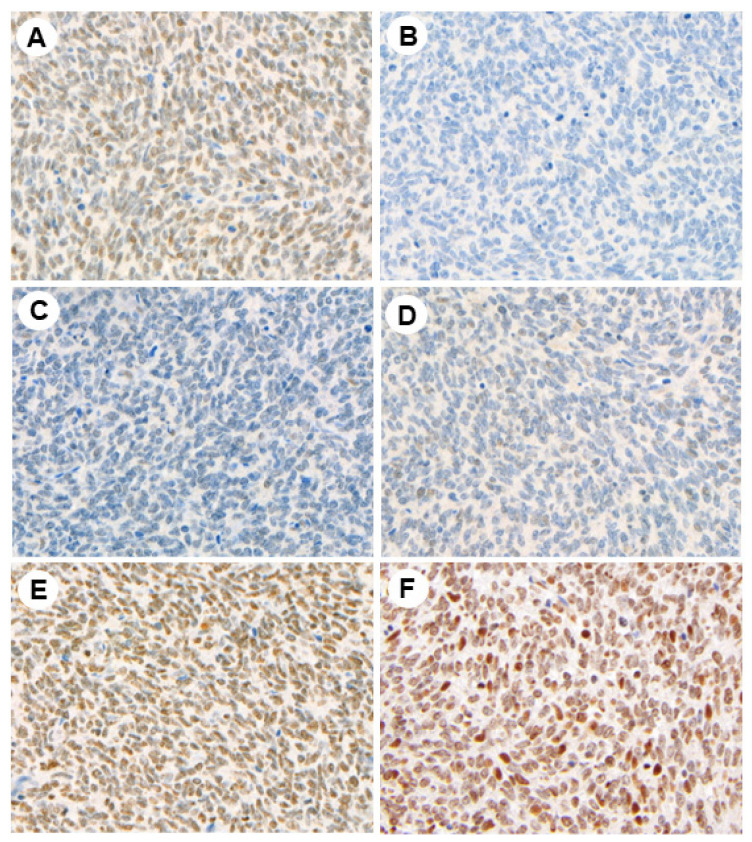
Immunohistochemical expression of PRAME (**A**), TET1 (**B**), DNMT3A (**C**), DNMT3B (**D**), 5hmC (**E**), and H3ac (**F**) in synovial sarcoma (magnification ×200).

Representative photomicrographs of the IHC studies are shown in Figure 1, Figure 2 and Figure 3.

**Figure 4:**
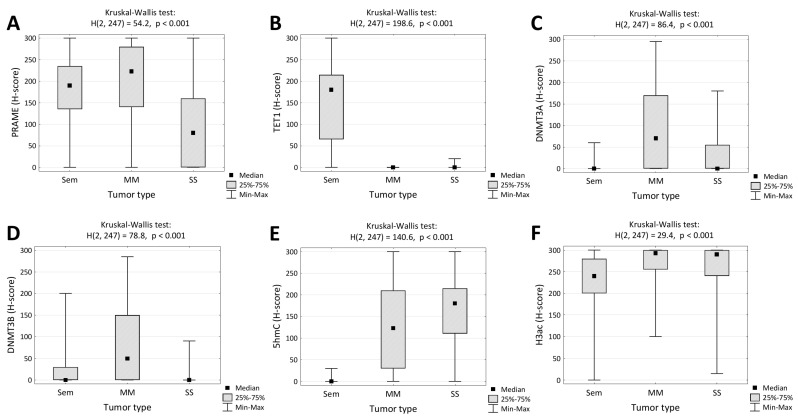
Quantitative analysis of expression of PRAME (**A**), TET1 (**B**), DNMT3A (**C**), DNMT3B (**D**), 5hmC (**E**), and H3ac (**F**) in testicular seminomas (Sem), mucosal melanomas (MM), and synovial sarcomas (SS).

All three tumor types were characterized by a wide spectrum of PRAME expressions ranging from negative (h-score = 0) to strong and diffuse (h-score = 300). PRAME h-scores of >10 (positive expression) were observed in 97% (77/79) seminomas, 94% (62/66) sinonasal melanomas, and 74% (75/102) synovial sarcomas. Median PRAME expression was highest in melanomas and lowest in synovial sarcomas (Figure 4A). 

H-score values were comparable in seminomas and seminoma components of mixed GCT (Supplemental Figure S2). TET1 immunopositivity was present in 90% (71/79) of seminomas, and 2% (2/102) of synovial sarcomas had faint immunoreactivity and all melanomas were negative (Figure 4B). Conversely, melanomas revealed frequent expression of methyltransferases DNMT3A (49/66, 74%) and DNMT3B (44/66, 67%). However, expression of these enzymes was remarkably lower in seminomas and synovial sarcomas. DNMT3A was expressed in 3% (2/79) of seminomas and 36% (37/102) of synovial sarcomas, while DNMT3B was expressed in 33% (27/79) of seminomas and 5% (5/102) of synovial sarcomas (Figure 4C,D). Subsequently, the expression of 5hmC, an intermediate in DNA demethylation, and the pan-acetylation of histone 3 were evaluated. Weak 5hmC immunostaining was seen in 3% of seminomas (2/79). In contrast, 80% of melanomas (53/66) and 95% of synovial sarcomas (97/102) had positive 5hmC IHC (Figure 4E). H3ac was detected in all analyzed tumors, usually with high h-scores (Figure 4F).

### 3.2. Correlations between Expression of PRAME, Methylation Regulators and Epigenetic Marks

Kendall’s tau correlations for the studied tumor groups are summarized in Table 1. In all tumor categories, PRAME expression correlated with increased histone 3 acetylation. Also, PRAME IHC positively correlated with both DNMTs and DNMT3A in melanomas and synovial sarcomas, respectively. A positive correlation between PRAME and TET1 expression was seen in seminomas. However, no similar correlation was seen between expression of PRAME and 5hmC, a product of 5mC oxidation by TET enzymes.

## 4. Discussion

PRAME is a promising target for cancer immunotherapy. Recent and ongoing clinical trials have employed various techniques, including peptide vaccines, T cell-engaging molecules, and adoptive transfer of engineered T cells that activate the immune system against PRAME-expressing tumor cells. Those drugs have been tested in solid tumors and hematological malignancies (ClinicalTrials.gov). An iatrogenic enhancement of PRAME expression in cancer cells may improve the efficacy and applicability of such novel treatments. Several previous studies evidenced that PRAME is regulated epigenetically through the gene promotor methylation and histone acetylation [10,11,15,21]. 

This study employed IHC to evaluate the expression of key methylating/demethylating enzymes (DNMT3A, DNMT3B, and TET1) and two epigenetic marks, namely, 5-hydroxymethylcytosine and histone 3 acetylation. Tumors from three different lineages (sinonasal melanoma, testicular seminoma, and synovial sarcoma), identified to have common yet variable PRAME immunoreactivity, were studied [1]. 

In seminomas, expression of DNMT3A or DNMT3B was at a low level, as previously reported [22,23]. However, it was paired with elevated levels of demethylase TET1 and low expression of 5hmC (2/79, 3%). The latter likely reflects genomic hypomethylation and a shortage of substrate (5mC) for oxidation [22]. A recent study reported differential expression of PRAME in seminomas and seminoma components of mixed tumors [24]. This disparity is believed to result from PRAME involvement in the “reprogramming” of seminoma cells towards the embryonal carcinoma phenotype [24]. Almost all testicular embryonal carcinomas lack PRAME expression [1,15,24]. In our cohort, median PRAME h-scores for seminomas and seminoma components of mixed germ cell tumors were not statistically different (Supplemental Figure S2). 

In synovial sarcomas, a positive association between expression of PRAME and DNMT3A was identified. Also, PRAME staining was strongly, positively correlated with global 5hmC levels. However, TET1 expression was infrequent. This might imply involvement of other enzymes in the oxidation of 5-methylcytosine in these tumors. Additional studies are required to address the nature and significance of this association.

In melanoma, positive associations between expression of PRAME and DNMT3A and DNMT3B were seen. Both DNMT3A and DNMT3B manifest oncogenic properties in melanoma and have been linked with unfavorable clinical outcomes [25,26,27]. Increased PRAME expression in melanomas has also been linked with poor prognosis, and this might explain the co-occurrence of this marker with DNMTs [28,29].

Increased histone acetylation was associated with higher PRAME expression. These findings corroborate earlier experimental observations that PRAME may be induced by histone deacetylase inhibitors (HDACIs) [15,16]. Two embryonal carcinoma cell lines, 2102EP and NCCIT, which are naturally PRAME-negative and hypermethylated, revealed upregulated PRAME expression following treatment with various HDACIs [15]. Thus, a formation of euchromatin around the PRAME locus via histone deacetylation appears to override the repressive DNA methylation mark within the gene promoter, allowing for de-repression of PRAME expression [15]. Functional link between histone acetylation and PRAME is further supported by a recent study on laryngeal squamous cell carcinoma in which the expression of PRAME was negatively correlated with IHC staining for histone deacetylase 5 [7]. Nevertheless, validation of these observations will require further studies on fresh/frozen tissues, employing techniques allowing for protein quantitation and measurements of enzymatic activities.

Overall, apart from a weak correlation between TET1 and PRAME expression in seminomas, we found no evidence supporting a relationship between PRAME and the global expression/activities of DNA methyltransferases DNMT3A/B or demethylase TET1 in the analyzed tumors. However, histone acetylation might be a common mechanism regulating the transcription of PRAME in distinct types of cancer. Therefore, the addition of HDACIs to anti-PRAME immunotherapy might improve its clinical efficacy through increased expression and presentation of PRAME antigens. 

HDACIs themselves are an emerging class of anticancer compounds acting through diverse downstream mechanisms, e.g., induction of differentiation, apoptosis, growth arrest, or inhibition of neoangiogenesis. Promising effects of HDACIs have been shown in a number of tumors, including melanoma, testicular germ cell tumors, and sarcomas [30,31,32].

## 5. Conclusions

Despite PRAME expression in cancer presumably being regulated by gene methylation, we found no relevant, direct correlation with DNA methylase/demethylase expression. Regulatory cues that influence PRAME methylation might therefore be more site-specific. On the other hand, global levels of H3ac were positively correlated with PRAME in three distinct types of cancer: seminoma, synovial sarcoma, and melanoma. Although histone acetylation, as a determinant of PRAME expression, warrants further studies, there may be a rationale for the use of anti-PRAME immunotherapy in combination with histone deacetylase inhibitors in cancer treatment.

## Figures and Tables

**Table 1:** Kendall’s tau correlation coefficient values for the IHC expression of PRAME and methylation regulators TET1 and DNMT3A/B, and epigenetic marks 5hmC and H3ac.

	Kendall’s tau	*p* Value
All (n = 247)		
TET1	**0.206**	**<0.001**
DNMT3A	**0.229**	**<0.001**
DNMT3B	**0.329**	**<0.001**
5hmC	−0.033	0.408
H3ac	**0.176**	**<0.001**
Seminoma (n = 79)		
TET1	**0.246**	**0.001**
DNMT3A *	−0.158	0.040
DNMT3B	−0.010	0.894
5hmC *	−0.202	0.009
H3ac	**0.300**	**<0.001**
Mucosal melanoma (n = 66)		
TET1	NA	NA
DNMT3A	**0.316**	**<0.001**
DNMT3B	**0.347**	**<0.001**
5hmC	0.100	0.235
H3ac	**0.188**	**0.026**
Synovial sarcoma (n = 102)		
TET1 *	0.108	0.109
DNMT3A	**0.338**	**<0.001**
DNMT3B *	0.182	0.007
5hmC	**0.421**	**<0.001**
H3ac	**0.299**	**<0.001**

* false correlations—insufficient number of tumors with positive IHC; statistically significant correlations are in bold text.

## Data Availability

The datasets generated and analyzed in this study are available on reasonable request from the corresponding author.

## Supplementary Materials

The following supporting information can be downloaded at https://www.mdpi.com/article/10.3390/jcm13061554/s1: Figure S1: H&E-stained sections of representative examples of the studied tumors: testicular seminoma (A,B), mucosal melanoma (C,D), synovial sarcoma (E,F) (magnification ×200); Figure S2: Quantitative analysis and comparison of PRAME expression in pure seminomas and seminoma components of mixed germ cell tumors; Table S1: Detailed information about antibodies and immunohistochemical protocols.
