# Supplementary material for: Expression of Immunotherapy Target PRAME in Cancer Correlates with Histone H3 Acetylation and Is Unrelated to Expression of Methylating (DMNT3A/3B) and Demethylating (TET1) Enzymes
Source: J Clin Med. 2024 Mar 8;13(6):1554. doi: 10.3390/jcm13061554 (PMC10971184; doi:10.3390/jcm13061554)
Supplement: Supplementary file 1 [file jcm-13-01554-s001.zip › jcm-2880516-supplementary Table S1.pdf]

Supplemental Table S1. Detailed information about antibodies and immunohistochemical protocols.

Antigen ID	Host and clonality	Clone/product ID	Vendor	Dilution	Positive controls	Procedure/visualisation	Platform
PRAME	rabbit monoclonal	EPR20330	Abcam	1:1000	melanoma, endometrium	20’ High pH EnVision Target Retrieval; EnVision Peroxidase/DAB Detection	DAKO Autostainer Link 48
TET1	mouse monoclonal	GT1462	Termo Fisher Scientific	1:500	testis	20’ High pH EnVision Target Retrieval; EnVision Peroxidase/DAB Detection	DAKO Autostainer Link 48
DNMT3A	rabbit monoclonal	D23G1	Cell Signaling Technology	1:50	placenta, testis	20’ High pH EnVision Target Retrieval; overnight incubation with primary antibody; EnVision Peroxidase/DAB Detection	DAKO Autostainer Link 48
DNMT3B	rabbit polyclonal	ab227883	Abcam	1:100	lung squamous cell ca, testis	20’ High pH EnVision Target Retrieval; EnVision Peroxidase/DAB Detection	DAKO Autostainer Link 48
5hmC	rabbit polyclonal	#39769	Active Motif	1:10000	brain	20’ High pH EnVision Target Retrieval; EnVision Peroxidase/DAB Detection	DAKO Autostainer Link 48
H3ac	rabbit polyclonal	PA5-114693	Thermo Fisher Scientific	1:200	colorectal cancer, brain	20’ Leica Bond ER1 Retrieval; Bond Polymer Refine Detection	Leica Bond-Max
