# Supplementary figures and images for: Expression of Immunotherapy Target PRAME in Cancer Correlates with Histone H3 Acetylation and Is Unrelated to Expression of Methylating (DMNT3A/3B) and Demethylating (TET1) Enzymes
Source: J Clin Med. 2024 Mar 8;13(6):1554. doi: 10.3390/jcm13061554 (PMC10971184; doi:10.3390/jcm13061554)

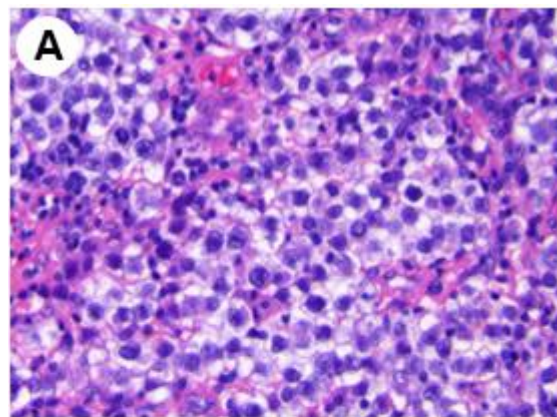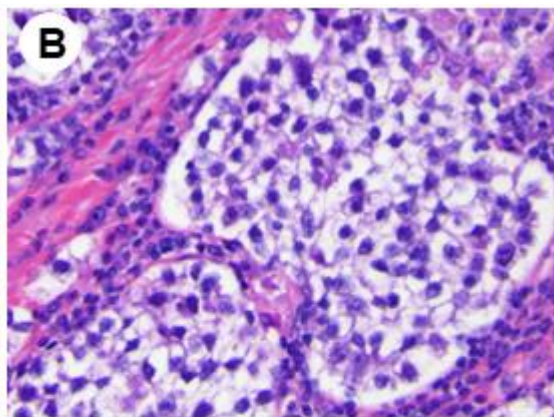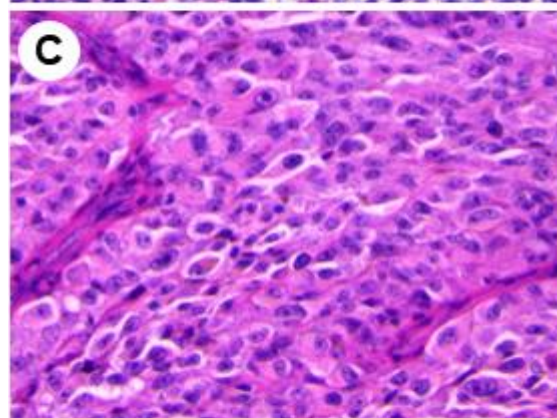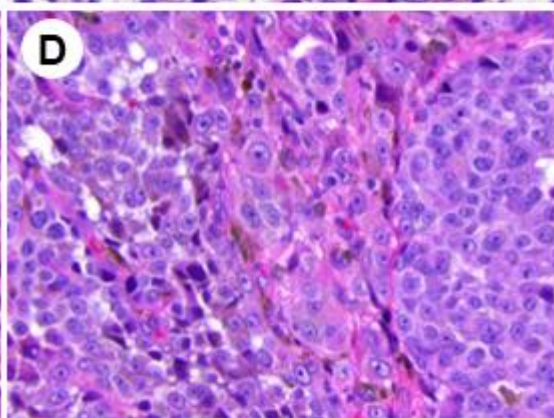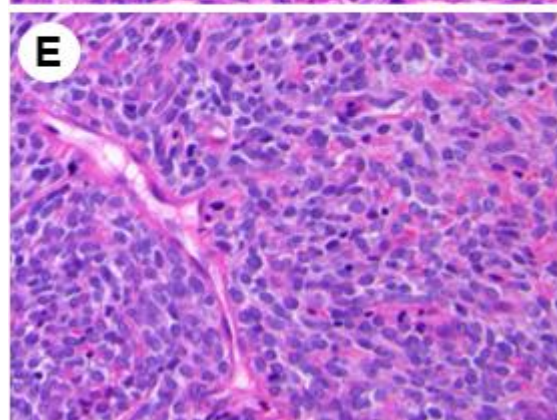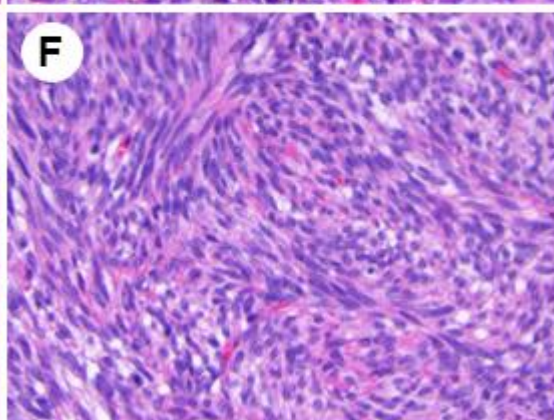

Supplement: Supplementary file 1 [file jcm-13-01554-s001.zip › jcm-2880516-supplementary Figure S1.pdf]

Mann-Whitney U test  
 $Z = -0.726, p = 0.468$

■ Median  
▨ 25%-75%  
└─┘ Min-Max

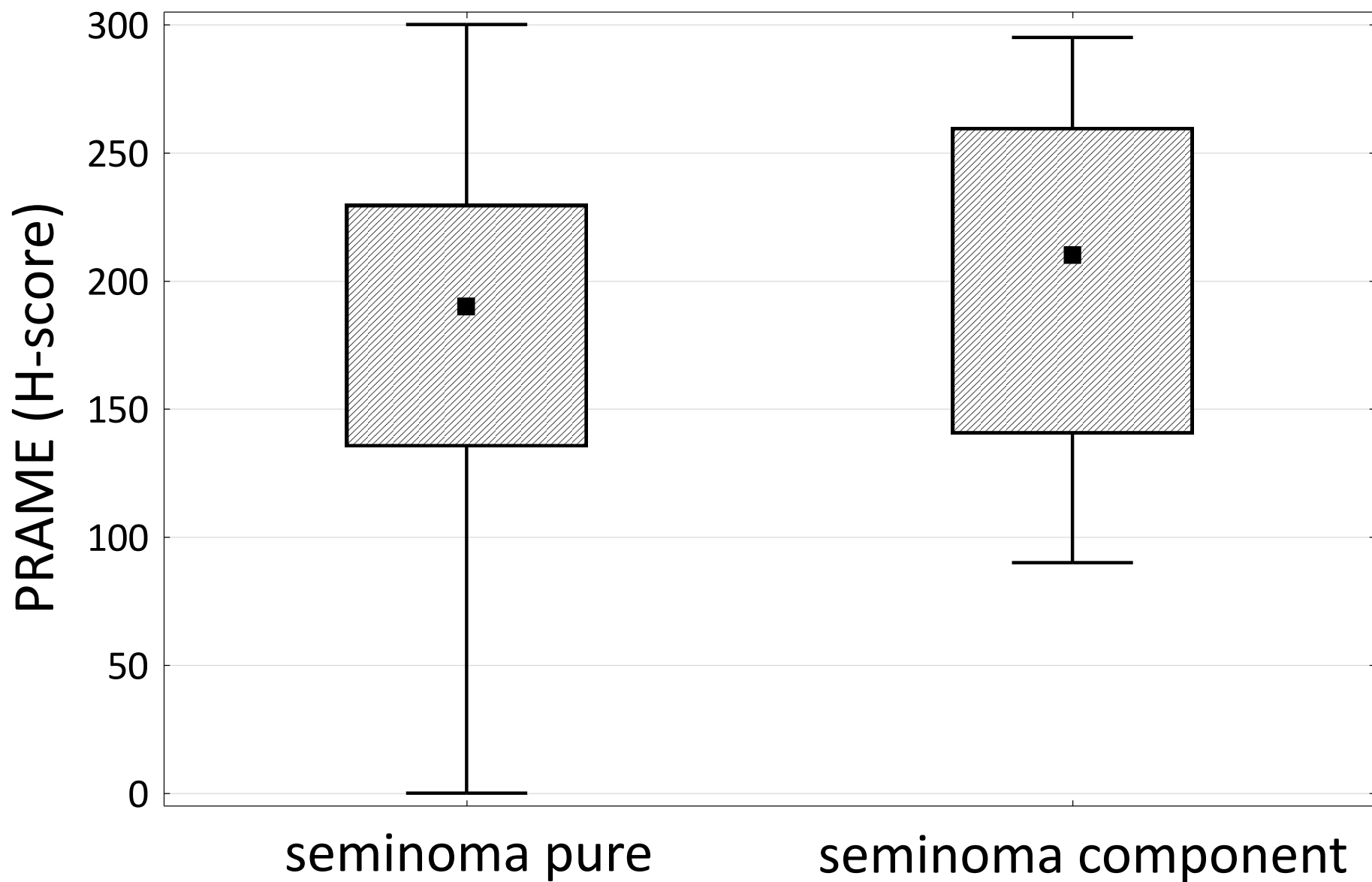

Supplement: Supplementary file 1 [file jcm-13-01554-s001.zip › jcm-2880516-supplementary Figure S2.pdf]
